# Equilibrium phase contrast-enhanced magnetic resonance angiography of the thoracic aorta and heart using balanced T1 relaxation-enhanced steady-state

**DOI:** 10.1016/j.jocmr.2024.101046

**Published:** 2024-05-27

**Authors:** Robert R. Edelman, Onural Ozturk, Amit Pursnani, Senthil Balasubramanian, Nondas Leloudas, Ioannis Koktzoglou

**Affiliations:** a Department of Radiology, Northshore University HealthSystem, Evanston, Illinois, USA; b Department of Radiology, Feinberg School of Medicine, Northwestern University, Chicago, Illinois, USA; c Department of Medicine, Pritzker School of Medicine, University of Chicago, Chicago, Illinois, USA; d Department of Radiology, Pritzker School of Medicine, University of Chicago, Chicago, Illinois, USA

**Keywords:** Balanced T1 relaxation-enhanced steady-state, Breath-hold, Electrocardiographic (ECG) gating, Magnetic resonance, MR angiography, Cardiac

## Abstract

**Background:**

Three-dimensional (3D) contrast-enhanced magnetic resonance angiography (CEMRA) is routinely used for vascular evaluation. With existing techniques for CEMRA, diagnostic image quality is only obtained during the first pass of the contrast agent or shortly thereafter, whereas angiographic quality tends to be poor when imaging is delayed to the equilibrium phase. We hypothesized that prolonged blood pool contrast enhancement could be obtained by imaging with a balanced T1 relaxation-enhanced steady-state (bT1RESS) pulse sequence, which combines 3D balanced steady-state free precession (bSSFP) with a saturation recovery magnetization preparation to impart T1 weighting and suppress background tissues. An electrocardiographic-gated, two-dimensional-accelerated version with isotropic 1.1-mm spatial resolution was evaluated for breath-hold equilibrium phase CEMRA of the thoracic aorta and heart.

**Methods:**

The study was approved by the institutional review board. Twenty-one subjects were imaged using unenhanced 3D bSSFP, time-resolved CEMRA, first-pass gated CEMRA, followed by early and late equilibrium phase gated CEMRA and bT1RESS. Nine additional subjects were imaged using equilibrium phase 3D bSSFP and bT1RESS. Images were evaluated for image quality, aortic root sharpness, and visualization of the coronary artery origins, as well as using standard quantitative measures.

**Results:**

Equilibrium phase bT1RESS provided better image quality, aortic root sharpness, and coronary artery origin visualization than gated CEMRA (P < 0.05), and improved image quality and aortic root sharpness versus unenhanced 3D bSSFP (P < 0.05). It provided significantly larger apparent signal-to-noise and apparent contrast-to-noise ratio values than gated CEMRA and unenhanced 3D bSSFP (P < 0.05) and provided ninefold better fluid suppression than equilibrium phase 3D bSSFP. Aortic diameter and main pulmonary artery diameter measurements obtained with bT1RESS and first-pass gated CEMRA strongly correlated (P < 0.05).

**Conclusions:**

We found that using bT1RESS greatly prolongs the useful duration of blood pool contrast enhancement while improving angiographic image quality compared with standard CEMRA techniques. Although further study is needed, potential advantages for vascular imaging include eliminating the current requirement for first-pass imaging along with better reliability and accuracy for a wide range of cardiovascular applications.

## Graphical abstract

**Figure:**
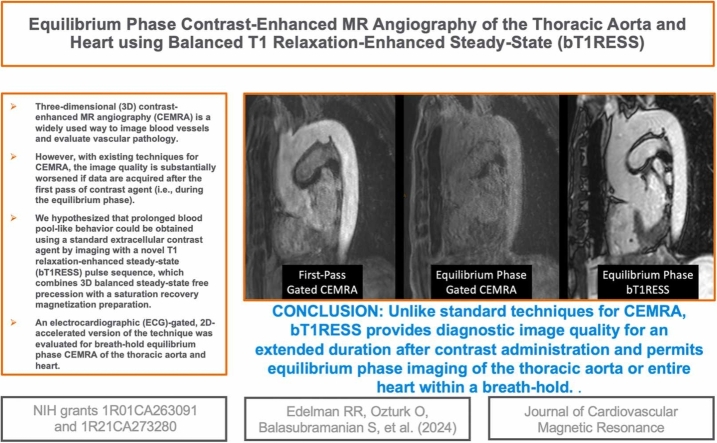

## 1 Background

Three-dimensional (3D) contrast-enhanced magnetic resonance angiography (CEMRA) is a valuable cross-sectional imaging tool to depict arterial anatomy and detect vascular pathology. Compared with digital subtraction angiography, magnetic resonance angiography (MRA) is noninvasive while compared with computed tomography (CT) angiography, it avoids the risks of iodinated contrast media and radiation. With CEMRA, data are acquired during the first pass of an extracellular gadolinium-based contrast agent (GBCA) coinciding with peak intravascular signal enhancement from T1 shortening of the blood pool [1]. Examples of CEMRA techniques include time-resolved CEMRA, which permits dynamic evaluation of vascular enhancement patterns [2], [3], [4], and electrocardiographic (ECG)-gated CEMRA, which is a useful technique for evaluating the aorta root [5], [6] and provides reproducible diameter measurements [7].

However, for both CEMRA and CT angiography, diagnostic image quality is only obtained during the first pass of the contrast agent or shortly thereafter, whereas angiographic quality tends to be poor when imaging is delayed to the equilibrium phase. Unfortunately, there are no second chances with these angiographic techniques. If something goes awry during a first-pass scan (e.g., the central k-space views are mistimed with respect to the contrast agent bolus, the intravenous line extravasates, or the patient moves or fails to adequately breath-hold), then the images may become nondiagnostic [8]. A potential solution is to use a blood pool contrast agent, such as ferumoxytol instead of an extracellular agent [9]. However, ferumoxytol has drawbacks, such as high cost and a black box warning from the United States Food and Drug Administration. Moreover, it is not a suitable contrast agent to image late gadolinium enhancement or first-pass myocardial perfusion, both key components of cardiovascular magnetic resonance (CMR) protocols in adults.

Recently, an imaging technique called balanced T1 relaxation-enhanced steady-state (bT1RESS) was described for contrast-enhanced magnetic resonance imaging (MRI) of brain tumors [10]. It consists of a 3D balanced steady-state free precession (bSSFP) pulse sequence that incorporates a saturation recovery magnetization preparation to impart T1 weighting and suppress signals from background tissues. bT1RESS has been shown to provide significantly better tumor conspicuity than standard 3D spoiled gradient-echo pulse sequences. Moreover, we have observed anecdotally that bT1RESS also improves the conspicuity of the intracranial vasculature. Based on these observations, we hypothesized that it might be possible to use bT1RESS to extend the usable time window for CEMRA well beyond that provided by existing techniques, which are currently limited to imaging during the first pass or for a short time thereafter. To test this hypothesis, we evaluated a breath-hold, two-dimensional (2D)-accelerated, ECG-gated version of bT1RESS with isotropic 1.1-mm spatial resolution in a cohort of subjects undergoing CMR and compared it with pre- and post-contrast 3D bSSFP and standard CEMRA techniques for evaluation of the thoracic aorta. We also tested the feasibility of using equilibrium phase bT1RESS for breath-hold whole-heart imaging.

## 2 Methods

### 2.1 Study cohort

The study was approved by the hospital's institutional review board. Waiver of consent was obtained for patients undergoing a clinically indicated cardiac MR exam. Imaging was performed at 1.5T (MAGNETOM Avanto Dot or Aera, Siemens Healthcare, Erlangen, Germany). Two patient cohorts (as described further on) were comprised of a total of 30 adult subjects including 1 healthy adult volunteer and 29 patients. Patient demographics are summarized in Table 1. In addition, one patient with a left atrial mass, one patient with a right atrial thrombus, one patient with an ascending aortic aneurysm, and one patient with a repaired type A aortic dissection were imaged with a subset of the protocol sequences, so were not included in the data analysis. A total dose of 0.15 mmol/kg of gadobutrol (Bayer Healthcare, Berlin, Germany) was administered.

**Table 1:** Patient demographic data (combined cohorts 1 and 2).

Characteristic	All patients (n = 29)
Age (years)	60.6 ± 16.9
Female n (%)	18 (62%)
BMI (kg/m^2^)	26.1 ± 4.9
GFR <60 mL/min	5 (17%)
Hypertensive	14 (48%)
Diabetic	4 (14%)
Hypercholesterolemic	14 (48%)
Smoking history	13 (45%)
Coronary artery disease	12 (41%)
Peripheral artery disease	3 (10%)

*BMI* body mass index, *GFR* glomerular filtration rate.

### 2.2 bT1RESS pulse sequence

**Fig. 1:**
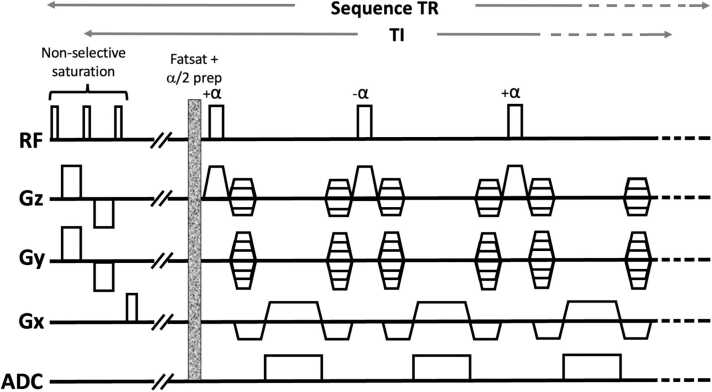
bT1RESS pulse sequence diagram. A magnetization preparation consisting of three 90° RF pulses is followed by a waiting period, fat saturation, and α/2 RF pulse. TI denotes the time between the saturation preparation and center of k-space. Data are collected using a high-bandwidth 3D bSSFP readout (simplified to show only three echoes). *RF* radiofrequency, *bT1RESS* balanced T1 relaxation-enhanced steady-state, *3D* three-dimensional, *bSSFP* balanced steady-state free precession, *ADC* analog-to-digital converter, *Gx* x-gradient, *Gy* y-gradient, Gz z-gradient, *TR* repetition time, *TI* time interval.

The pulse sequence diagram for bT1RESS is shown in Fig. 1. A composite saturation recovery magnetization preparation consisting of three 90° radiofrequency (RF) pulses is applied before a fat-suppressed 3D bSSFP readout. A single α/2 RF pulse is applied immediately before each diastolic-gated shot to drive the transverse magnetization toward the steady state. A user-selected TI value determines the time from the magnetization preparation to the center of k-space along the phase-encoding direction and includes the time to apply the fat saturation and α/2 preparation RF pulses. Based on prior experience using bT1RESS in the brain, the TI value was set to 320 ms, which was found effective at suppressing signals from cerebrospinal fluid and other background tissues. To minimize the breath-hold duration while maintaining sufficient slice coverage to span the thoracic aorta, 2D parallel acceleration was obtained using a generalized autocalibrating partially parallel acquisition [11]. Separate reference lines were acquired at the start of each scan (64 phase-encoding reference lines and 24 slice-encoding reference lines). Most imaging parameters for unenhanced 3D bSSFP were the same as bT1RESS except for the magnetization preparation. Reconstructed images were interpolated in both the 3D partition-encoding and in-plane directions.

### 2.3 Contrast-enhanced magnetic resonance angiography

Gadobutrol was diluted twofold by saline before infusion. Time-resolved CEMRA was acquired using 0.05 mmol/kg of gadobutrol infused at 4 mL/s. Next, first-pass ECG-gated CEMRA was acquired using 0.1 mmol/kg of gadobutrol infused at 2 mL/s, with scan timing determined from fluoroscopic triggering or the preceding time-resolved CEMRA acquisition depending on technologist preference. One 3D partition is acquired per heartbeat, with the number of heartbeats equal to the number of acquired 3D partitions. Imaging parameters for equilibrium phase gated CEMRA were identical to those used for the first-pass scan. Scan parameters for the MRA sequences are summarized in Table 2. For imaging of the aorta, CEMRA and bT1RESS were acquired in an oblique sagittal orientation using breath-holding. In some subjects, additional bT1RESS images were obtained in a three-chamber view for whole-heart imaging.

**Table 2:** Scan parameters for TWIST, gated CEMRA, and bT1RESS.

	TWIST	Gated CEMRA	bT1RESS
Acquired voxel size (mm)	2.29 × 1.38 × 2.54	1.35 × 1.15 × 2.92	2.19 × 2.19 × 2.20
Reconstructed voxel size (mm)	1.38 × 1.38 × 1.80	1.15 × 1.15 × 1.80	1.09 × 1.09 × 1.10
Slice oversampling (%)	0	8.3	50
Field of view (mm)	420	450	385
# slices (acquired/reconstructed)	56	48	64
Partial Fourier (phase, slice)	6/8, 6/8	7/8, 6/8	7/8, 7/8
Asymmetric echo	Yes	Yes	No
Parallel acceleration	GRAPPA 2	GRAPPA 2	GRAPPA 2 × 2
TR/TE/TI (ms)	2.40/0.85/none	2.75/0.94/none	2.90/1.45/320
Excitation flip angle (deg)	25	40	70
Magnetization preparation	No	No	Composite saturation, fat suppression
Electrocardiographic gating	No	Yes	Yes
Shot duration (ms)	n/a	286	186
Trigger delay (ms)	n/a	250	300
Bandwidth (Hz/pixel)	650	590	1495
Readout	3D spoiled gradient-echo	3D spoiled gradient-echo	3D bSSFP
Image subtraction	Yes	No	No
Other	Central region A 33%, sampling density B 50%, 25 measurements	Contrast agent timing obtained using prior TWIST or fluoro triggering	Local shimming to heart and aorta

*TWIST* time-resolved angiography with interleaved stochastic trajectories*, CEMRA* contrast-enhanced magnetic resonance angiography*, bT1RESS* balanced T1 relaxation-enhanced steady-state*, GRAPPA* generalized autocalibrating partially parallel acquisition*, 3D* three-dimensional, *bSSFP* balanced steady-state free precession*, TR* repetition time, *TE* echo time, *TI* time interval.

Two cohorts of subjects were studied. Cohort 1 consisted of 21 subjects in whom 7 angiographic sequences were compared (unenhanced 3D bSSFP, time-resolved angiography with interleaved stochastic trajectories (TWIST) CEMRA, first-pass gated CEMRA, early (≈4 to 7 minutes post-contrast) and late (≈10 to 15 minutes post-contrast) equilibrium phase gated CEMRA and bT1RESS), except for 2 subjects in whom first-pass gated CEMRA was not acquired. Cohort 2 consisted of nine subjects in whom angiographic imaging was performed with two sequences (equilibrium phase 3D bSSFP and equilibrium phase bT1RESS at approximately 4 minutes post-contrast).

### 2.4 T1 and T2 mapping

In nine subjects, T1 and T2 maps were obtained in the mid short axis before and ≈6 to 10 minutes after contrast infusion. T1 maps were acquired using a modified look-locker inversion-recovery sequence, while T2 maps were acquired using a T2-prepared bSSFP sequence [12] per our standard clinical cardiac MR protocol.

### 2.5 Qualitative image analysis

Images were reviewed by three readers with 2, 3, and >10 years of experience, respectively, interpreting CMR studies. Image quality was scored subjectively according to a 4-point scale: (1) nondiagnostic, aortic wall not assessable due to artifacts; (2) fair, with moderate artifacts; (3) good, with mild artifacts; and (4) excellent image quality with negligible artifacts.

### 2.6 Quantitative image analysis

Region-of-interest analysis was performed using an Intelerad picture archiving and communication system (PACS) workstation (Montreal, Quebec, Canada). Tissue signal and standard deviation were measured in the air anterior to the chest wall, subcutaneous fat, chest muscles, mid-ascending aorta, and superior vena cava. Since parallel acceleration was used, the true signal-to-noise ratio cannot be determined without additional measurements [13]. Therefore, we refer to the apparent signal-to-noise ratio (aSNR), defined as tissue signal 0.655 × S/σ_air_, and the apparent contrast-to-noise ratio (aCNR) between two tissues, defined as 0.655 × (S_1_ – S_2_)/σ_air_. For comparisons of equilibrium phase 3D bSSFP and bT1RESS in cohort 2, signal intensity ratios (computed as the signal for bT1RESS divided by the signal for 3D bSSFP) were measured using regions-of-interest in the ascending aorta, subcutaneous fat, chest muscles, and cerebrospinal fluid. Diameter measurements for first-pass gated CEMRA (which was used as the internal reference standard) and equilibrium phase bT1RESS were performed at the level of the sinuses of Valsalva, sinotubular junction, mid-ascending aorta, descending aorta, and main pulmonary artery.

### 2.7 Statistical analysis

Due to non-normality, qualitative and quantitative data were analyzed using non-parametric Friedman or Wilcoxon signed-rank tests. Quantitative and qualitative measures obtained with 3D bSSFP and bT1RESS were compared to the same phase gated CEMRA (if available), and to time-resolved CEMRA and first-pass gated CEMRA measures. Inter-rater agreement was assessed using quadratically-weighted Gwet’s AC2 statistic (Gwet's AC1 was substituted for AC2 if the latter was not computable); values of 0.01–0.20, 0.21–0.40, 0.41–0.60, 0.61–0.80, and 0.81–0.99 were considered as slight, fair, moderate, substantial, and almost perfect, respectively. Pearson’s correlation was used to evaluate the correlation between vessel diameters; values of ≤0.30, 0.31–0.69, and ≥0.70 were interpreted to indicate weak, moderate, and strong correlations, respectively. Analyses were performed using R (version 4.2.2, The R Foundation for Statistical Computing, Vienna, Austria) or SPSS (version 22.0, IBM SPSS Statistics, Armonk, New York) software. P values less than 0.05 were considered statistically significant.

## 3 Results

Mean scan times for first-pass gated CEMRA and 4-minute bT1RESS (as determined from the Digital Imaging and Communications in Medicine image headers) were 24.4 seconds (range 19.3 to 37.0 seconds) and 18.4 seconds (range 14.1 to 28.2 seconds), respectively. Quantitative and qualitative results are summarized in Table 3. Image quality for 3D bSSFP and bT1RESS was, on average, good to excellent.

**Table 3:** Qualitative and quantitative data.

	*Image quality*	*Aortic root sharpness*	*Coronary artery origins*	*Aorta aSNR*	*Superior vena cava aSNR*	*Aorta-fat aCNR*	*Aorta-muscle aCNR*

Unenhanced 3D bSSFP	3.0 ± 0.4^a^	3.4 ± 0.4^a^^,^^b^	1.9 ± 0.2^a^^,^^b^	1059 ± 411^a^^,^^b^	963 ± 354^a^^,^^b^	116 ± 500	752 ± 340^a^^,^^b^
Early phase bT1RESS	3.8 ± 0.4^a^^,^^b^^,^^c^^,^^d^	3.8 ± 0.5^a^^,^^b^^,^^c^^,^^d^	1.8 ± 0.4^a^^,^^b^^,^^c^	1954 ± 792^a^^,^^b^^,^^c^^,^^d^	1763 ± 573^a^^,^^b^^,^^c^^,^^d^	689 ± 500^a^^,^^b^^,^^c^^,^^d^	1473 ± 65^a^^,^^b^^,^^c^^,^^d^
Late phase bT1RESS	3.7 ± 0.3^a^^,^^b^^,^^c^^,^^d^	3.7 ± 0.5^a^^,^^b^^,^^c^^,^^d^	1.8 ± 0.3^a^^,^^b^^,^^c^	1826 ± 875^a^^,^^b^^,^^c^^,^^d^	1748 ± 848^a^^,^^b^^,^^c^^,^^d^	495 ± 536^a^^,^^b^^,^^c^^,^^d^	1373 ± 677^a^^,^^b^^,^^c^^,^^d^
Time-resolved CEMRA	2.6 ± 0.5	2.1 ± 0.5	0.7 ± 0.6	97 ± 41	32 ± 15	−6.5 ± 46	83 ± 40
First-pass CEMRA	3.0 ± 0.6^a^	2.7 ± 0.6^a^	1.4 ± 0.5^a^	261 ± 100^a^	364 ± 148^a^	63.3 ± 75^a^	189 ± 100^a^
Early phase CEMRA	2.0 ± 0.5	1.9 ± 0.6	0.9 ± 0.6	177 ± 63	203 ± 73	−5.6 ± 47	103 ± 55
Late phase CEMRA	1.3 ± 0.4	1.4 ± 0.4	0.5 ± 0.3	150 ± 63	176 ± 53	−20.5 ± 41	81 ± 71
							
*Diameter measures*	*Sinus of valsalva (mm)*	*Sinotubular junction (mm)*	*Mid-ascending aorta (mm)*	*Descending aorta (mm)*	*Main pulmonary artery (mm)*		
Unenhanced bSSFP	34.7 ± 4.5	28.5 ± 4.3	32.7 ± 5.3	22.3 ± 2.6	25.2 ± 2.5^a^		
Early phase bT1RESS	34.8 ± 4.5	28.4 ± 3.8^c^	32.8 ± 5.1^c^	22.2 ± 2.8^c^	25.2 ± 2.6^a^		
Late phase bT1RESS	35.0 ± 4.6^d^	28.8 ± 3.7^c^	33.0 ± 5.4	22.3 ± 2.7^c^	25.1 ± 2.6^a^^,^^c^		
Time-resolved CEMRA	34.8 ± 4.2	28.9 ± 3.5	33.0 ± 5.1	22.1 ± 2.4	24.2 ± 2.7		
First-pass CEMRA	34.3 ± 5.2	29.1 ± 3.8	33.0 ± 5.2	22.1 ± 2.5	25.5 ± 2.5^a^		
Early phase CEMRA	35.2 ± 4.3	29.7 ± 4.0	33.5 ± 5.1	22.5 ± 2.6	25.7 ± 2.5		
Late phase CEMRA	35.7 ± 4.3	29.6 ± 3.6	33.6 ± 5.0	23.2 ± 3.3	26.2 ± 3.1		

Data presented as mean ± standard deviation. Image quality, aortic root sharpness, and visualized coronary artery origins are the average of three readers.

*aSNR* apparent signal-to-noise ratio, *aCNR* contrast-to-noise ratio, *3D* three-dimensional, *bSSFP* balanced steady-state free precession, *bT1RESS* balanced T1 relaxation-enhanced steady-state, *CEMRA* contrast-enhanced magnetic resonance angiography.

a P < 0.05 versus time-resolved CEMRA.

b P < 0.05 versus first-pass CEMRA.

c P < 0.05 versus same phase CEMRA.

d P < 0.05 versus unenhanced bSSFP.

In cohort 1, bT1RESS provided better image quality than CEMRA (P < 0.05, all comparisons), while both unenhanced 3D bSSFP and bT1RESS provided improved aortic root sharpness and number of visualized coronary origins over CEMRA (P < 0.05, all comparisons). Compared to unenhanced 3D bSSFP, equilibrium phase bT1RESS improved image quality, aortic root sharpness, and all aSNR and aCNR measures. Aortic and main pulmonary artery diameter measurements for unenhanced 3D bSSFP and equilibrium phase bT1RESS were similar in magnitude and showed a strong correlation with first-pass gated CEMRA (r = 0.87 at the level of the sinuses of Valsalva; r = 0.94 for the sinotubular junction, r = 0.98 for the mid-ascending aorta, r = 0.99 for the descending aorta, and r = 0.96 for the main pulmonary artery, respectively; P < 0.001 for all).

In cohort 2, comparing equilibrium phase 3D bSSFP and equilibrium phase bT1RESS, mean image quality scores were 3.4 and 3.9 (P < 0.01). Comparing equilibrium phase bT1RESS with equilibrium phase 3D bSSFP, the signal intensity ratios (mean ± sd) for aorta, skeletal muscle, subcutaneous fat, and cerebrospinal fluid were, respectively, 0.96 ± 0.06, 0.82 ± 0.34, 0.82 ± 0.13, and 0.11 ± 0.05.

Inter-reader agreement data are provided in Table 4. Inter-rater agreement for unenhanced 3D bSSFP as well as early and delayed phase bT1RESS was substantial to almost perfect for image quality, aortic root sharpness, and visualized coronary artery origins. Conversely, inter-rater agreement for CEMRA scans was slight to moderate for visualized coronary artery origins.

**Table 4:** Inter-reader agreement.

	Image quality	Aortic root sharpness	Visualized coronary artery origins
Unenhanced bSSFP	0.85 (0.74, 0.96)*	0.84 (0.71, 0.96)*	0.88 (0.76, 1.00)*
Early phase bT1RESS	0.98 (0.94, 1.00)*	0.96 (0.91, 1.00)*	0.90 (0.77, 1.00)*
Late phase bT1RESS	0.80 (0.68, 0.93)*	0.89 (0.76, 1.00)*	0.85 (0.68, 1.00)*
Time-resolved CEMRA	0.89 (0.85, 0.94)*	0.87 (0.81, 0.92)*	0.52 (0.22, 0.82)*
First-pass CEMRA	0.91 (0.86, 0.96)*	0.83 (0.76, 0.90)*	0.47 (0.16, 0.77)*
Early phase CEMRA	0.68 (0.59, 0.77)*	0.62 (0.46, 0.79)*	0.11 (−0.18, 0.40)†
Late phase CEMRA	0.85 (0.76, 0.95)*	0.84 (0.74, 0.93)*	0.60 (0.36, 0.84)*

Inter-reader agreement AC2 scores with 95% confidence intervals. ^†^AC1 value.

*bSSFP* balanced steady-state free precession*, bT1RESS* balanced T1 relaxation-enhanced steady-state*, CEMRA* contrast-enhanced magnetic resonance angiography*.*

* P < 0.05.

† AC1 value.

Measured T1 and T2 values (n = 9, expressed as mean ± sd) were as follows: pre T2 (LV cavity) = 190 ± 19 ms; pre T2 (myocardium) = 53 ± 4 ms; post T2 (LV cavity) = 147 ± 6 ms; post T2 (myocardium) = 61 ± 5 ms; pre T1 (LV cavity) = 1647 ± 82 ms; pre T1 (myocardium) = 1045 ± 53 ms; post T1 (LV cavity) = 179 ± 52 ms; post T1 (myocardium) = 305 ± 56 ms.

**Fig. 2:**
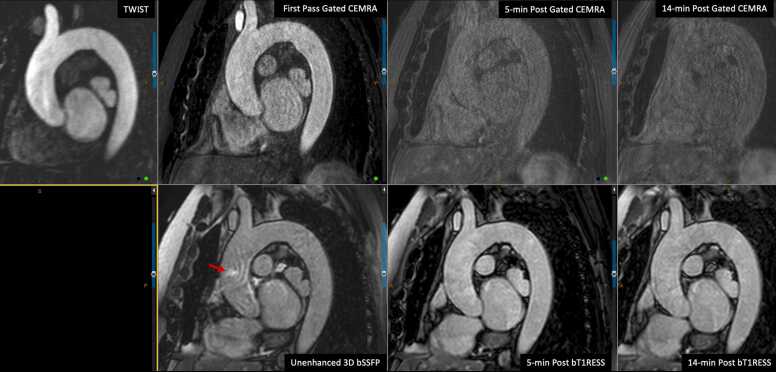
Patient with non-ischemic cardiomyopathy and severely reduced left ventricular ejection fraction of 20%. Pulse sequence comparisons using a candy cane view centered on the ascending aorta. Top row = standard CEMRA sequences (first-pass TWIST CEMRA; first-pass gated CEMRA; early (5-minutes) and late (14-minutes) equilibrium phase gated CEMRA). Bottom row: unenhanced 3D bSSFP; early and late equilibrium phase bT1RESS. While gated CEMRA shows poor angiographic image quality at 5 minutes after contrast administration, angiographic image quality remains excellent even at 14 minutes post-contrast using bT1RESS. Note that parallel imaging artifacts (arrow) seen in the aortic root with unenhanced 3D bSSFP are barely visible with bT1RESS. *TWIST* time-resolved angiography with interleaved stochastic trajectories, *CEMRA* contrast-enhanced magnetic resonance angiography, *bT1RESS* balanced T1 relaxation-enhanced steady-state, *3D* three-dimensional, *bSSFP* balanced steady-state free precession.

**Fig. 3:**
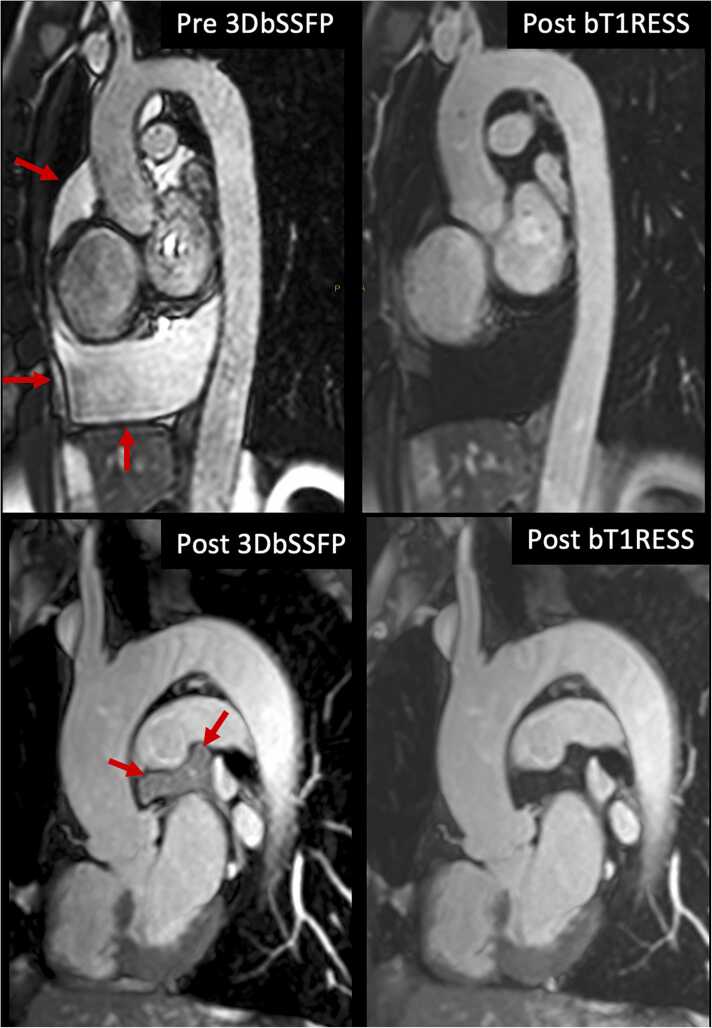
Comparison of 3D bSSFP and bT1RESS in two patients. Top row: a large pericardial effusion (arrows) appears bright with unenhanced 3D bSSFP (left) but is suppressed with equilibrium phase bT1RESS (right), improving the conspicuity of the ascending aorta and pulmonary vessels. Bottom row: equilibrium phase 3D bSSFP (left) shows undesirable background signal from pericardial fluid (red arrows) that is suppressed with bT1RESS (right). *bT1RESS* balanced T1 relaxation-enhanced steady-state, *3D* three-dimensional, *bSSFP* balanced steady-state free precession.

**Fig. 4:**
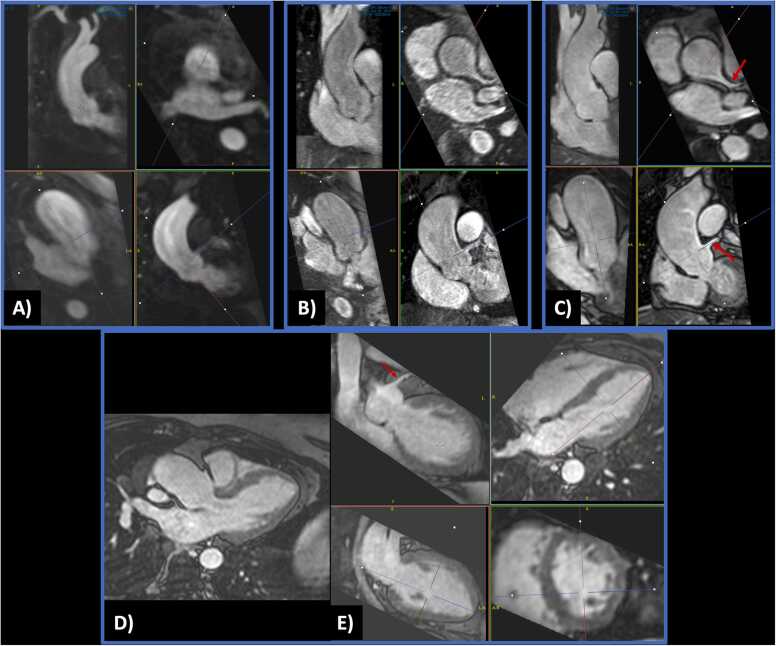
A 4.8-cm ascending aorta aneurysm. Comparison of multiplanar reconstructions from candy cane-oriented scans using (A) time-resolved TWIST CEMRA, (B) first-pass gated CEMRA, and (C) 5-minute post-contrast bT1RESS. Vessel sharpness and visualization of the left main coronary artery (arrow) are best with bT1RESS. Three-chamber bT1RESS (D) and corresponding orthogonal multiplanar reconstructions (E) show the dilated aortic root, left main coronary artery (arrow), and detailed cardiac anatomy. *TWIST* time-resolved angiography with interleaved stochastic trajectories, *CEMRA* contrast-enhanced magnetic resonance angiography, *bT1RESS* balanced T1 relaxation-enhanced steady-state.

**Fig. 5:**
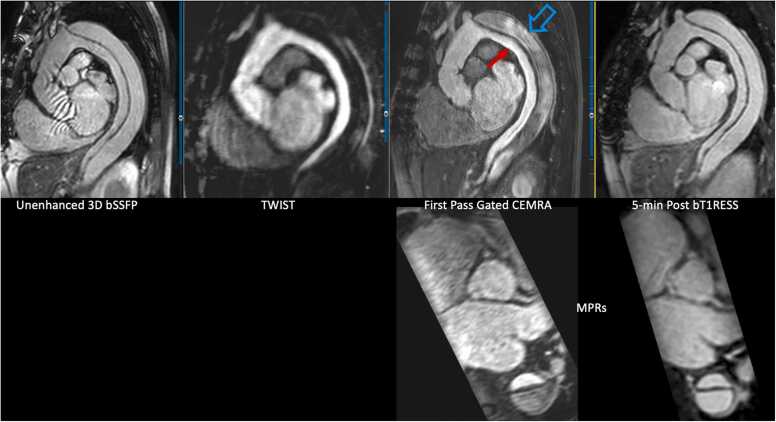
Repaired type A aortic dissection. Repaired type A aortic dissection. Red arrow = true lumen. Open blue arrow = false lumen. Top row, left-to-right: time-resolved TWIST CEMRA, first-pass gated CEMRA, 5-minute post-contrast bT1RESS. Bottom row: multiplanar reconstructions at the level of the sinuses of Valsalva for first-pass gated CEMRA and equilibrium phase bT1RESS. Early arterial frame from the TWIST CEMRA only shows the true lumen, consistent with slow flow in the false lumen. The dissection flap and false lumen are best visualized with equilibrium phase bT1RESS. *TWIST* time-resolved angiography with interleaved stochastic trajectories, *CEMRA* contrast-enhanced magnetic resonance angiography, *bT1RESS* balanced T1 relaxation-enhanced steady-state, *3D* three-dimensional, *bSSFP* balanced steady-state free precession.

**Fig. 6:**
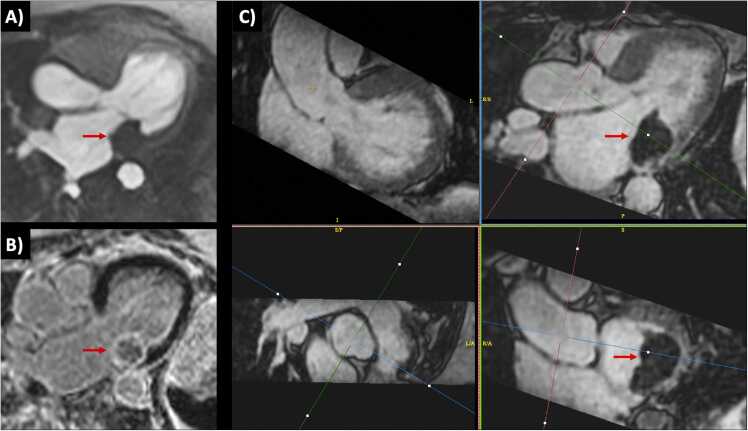
Patient with left atrial mass (arrows) discovered on echocardiography, likely caseous mitral annular calcification. (A) The lesion appears dark with first-pass perfusion imaging. (B) Late gadolinium enhancement image using single-shot inversion-recovery bSSFP shows mild peripheral enhancement. (C) The lesion is well depicted in multiplanar reconstructions from equilibrium phase bT1RESS. *bT1RESS* balanced T1 relaxation-enhanced steady-state, *bSSFP* balanced steady-state free precession.

**Fig. 7:**
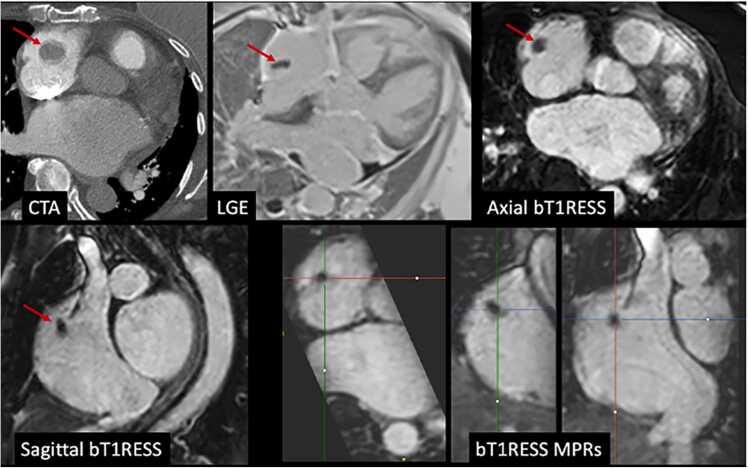
A 77-year-old female with 2-cm right atrial thrombus (arrow) on a CTA scan (top left) from 3 months prior. Late gadolinium enhancement image (top middle) obtained with standard 8-mm slice thickness during recent CMR shows that the thrombus has substantially decreased in size. Axial (top right) and sagittal (bottom left) equilibrium phase bT1RESS obtained with 1.1-mm × 1.1-mm × 1.1-mm isotropic voxels, along with corresponding multiplanar reconstructions (bottom right), show the 3D configuration of the thrombus and proximity to the cavo-atrial junction. *CTA* computed tomography angiography, *LGE* late gadolinium enhancement, *CMR* cardiovascular magnetic resonance, *bT1RESS* balanced T1 relaxation-enhanced steady-state, *3D* three-dimensional, *MPR* multiplanar reformation.

Angiographic image quality with bT1RESS was consistently better than unenhanced 3D bSSFP or equilibrium phase gated CEMRA. Fig. 2 provides a particularly striking example in a patient with dilated cardiomyopathy and poor cardiac function. Parallel imaging artifacts, when present, were less severe using equilibrium phase bT1RESS than unenhanced 3D bSSFP. With bT1RESS, suppression of pericardial fluid signal improved vessel visualization compared with unenhanced or equilibrium phase 3D bSSFP (Fig. 3). The use of isotropic spatial resolution permitted high-quality multiplanar reconstructions to be obtained (Fig. 4). Slow flow in the false lumen of an aortic dissection was well shown (Fig. 5), as were an intracardiac mass (Fig. 6) and thrombus (Fig. 7).

## 4 Discussion

We found that breath-hold equilibrium phase bT1RESS correlated closely with the internal reference technique (first-pass ECG-gated CEMRA) for depiction of the thoracic aorta, including the aortic root, and main pulmonary artery. bT1RESS provided better angiographic image quality, aortic root sharpness, and aSNR than unenhanced 3D bSSFP, first-pass, or equilibrium phase gated CEMRA. It also proved feasible to image a normal-sized heart in a single breath-hold by acquiring equilibrium phase bT1RESS in a three-chamber view.

Prolonged intravascular signal enhancement using 3D bSSFP was first described more than 20 years ago [14]. Whereas the T1 and T2 relaxivities of extracellular GBCA in blood are similar (e.g., r1 ≈ 5.2 L/[mmol*s]; r2 ≈ 6.1 L/[mmol*s] for gadobutrol) [15], there is a much larger decrease in the blood pool T1 compared with T2. This results in a large boost in the intravascular signal intensity when data are collected using a bSSFP readout, since the bSSFP signal intensity depends on the T2/T1 ratio [16]. In our study, the blood pool T2/T1 ratio was ≈0.12 pre-contrast versus ≈0.88 post-contrast. Blood pool aSNR was 12-fold better with bT1RESS versus gated CEMRA in the late equilibrium phase and 1.7-fold better versus unenhanced 3D bSSFP.

Comparing equilibrium phase bT1RESS with equilibrium phase 3D bSSFP, bT1RESS provided better image quality and more background suppression. Most importantly, it provided ninefold better suppression of fluid signal at the expense of a negligible 4% loss of blood pool signal. Robust suppression of background signals from pericardial fluid and other long T2 tissues is essential to avoid diagnostic confusion with enhancing blood vessels and to prevent overlap on maximum intensity projections.

### 4.1 Alternative approaches

Vascular imaging can be performed for certain indications using unenhanced free-breathing MRA techniques. For instance, unenhanced 3D bSSFP can be used to image the thoracic aorta [17], [18], [19], [20], [21], [22], [23], while a similar technique with a spatially-selective inversion preparation can be used for unenhanced renal artery imaging [24]. However, these techniques are relatively inefficient with typical scan times on the order of 5 to 15 minutes, and image quality can be degraded in patients with an irregular breathing pattern. Conversely, with equilibrium phase bT1RESS, the scan time is sufficiently short to permit breath-holding, while the short echo train duration of 186 ms is helpful to avoid artifacts from cardiac motion. These features were enabled through a combination of 2D parallel acceleration, single-shot echo train (i.e., all k_y_ lines for a given 3D partition were acquired in a single heartbeat), high sampling bandwidth, and short echo spacing. In our study, parallel imaging artifacts were usually minor and did not interfere with vessel evaluation. Moreover, artifacts were generally less conspicuous with equilibrium phase bT1RESS than unenhanced 3D bSSFP, presumably because of the higher intravascular signal and improved background suppression. More study is needed to explore the feasibility of using higher parallel acceleration factors to further improve temporal resolution or provide increased slice coverage.

Free-breathing, inversion-prepared CEMRA during the slow infusion of a contrast agent can be useful for whole-heart coronary artery MRA [25], [26], [27], [28]. However, this technique has not to our knowledge been used for angiographic imaging during the equilibrium phase of contrast enhancement. Instead of using an inversion preparation, we implemented equilibrium phase bT1RESS using a composite saturation recovery magnetization preparation. This has the theoretical benefit of resetting the longitudinal magnetization to zero at the start of each cardiac cycle, thereby minimizing any ghost artifacts caused by beat-to-beat signal variations from arrhythmias [29]. If an inversion preparation is used with bT1RESS, this benefit is lost, and a longer TI is needed to avoid blood pool signal suppression.

Compared with standard first-pass CEMRA techniques, there are several potential advantages of equilibrium phase bT1RESS. First, unlike first-pass CEMRA, equilibrium phase bT1RESS can be repeated as many times as needed. Second, images can be acquired in a single breath-hold, which is time-efficient and minimizes potential blurring from respiratory motion. Alternatively, a free-breathing implementation could be used to provide more flexibility with respect to temporal and spatial resolution [30]. Third, given the flexibility of the technique and excellent image quality for both arteries and veins, equilibrium phase bT_1_RESS could prove especially helpful for the evaluation of adult patients with congenital heart disease. For instance, it could be used to accurately depict an aortic coarctation or to delineate abnormal venous connections in partial anomalous pulmonary venous return. Fourth, by permitting angiographic images of diagnostic quality to be obtained many minutes after contrast administration, bT1RESS improves the visualization of slow flow compared with first-pass CEMRA and might better distinguish slow flow from thrombus. Finally, there is no downside to the technique since equilibrium phase bT1RESS is complementary to, rather than exclusive of, first-pass CEMRA. Moreover, it provides a robust failsafe in case there is a technically inadequate first-pass CEMRA [8].

## 5 Limitations

This study has limitations. Unlike first-pass CEMRA which predominantly shows arteries, equilibrium phase bT1RESS shows both arteries and veins, resulting in overlap on maximum intensity projection images. Second, a gadobutrol dose of 0.15 mmol/kg was administered, which is our standard dose for CMR exams. With a lower contrast agent dose or lower T1 relaxivity contrast agent, it might be necessary to lengthen the TI value to avoid suppressing the blood pool signal. Third, in our study, off-resonance effects (manifesting as banding artifacts and/or intravascular signal loss) were generally of no clinical consequence, likely due to the use of a very high sampling bandwidth and small voxels. However, off-resonance effects were often visible at the periphery of the field of view and sometimes caused signal loss in the left subclavian artery due to its proximity to the lung apex. Increasing the spatial resolution may worsen off-resonance artifacts if doing so requires the echo time to be lengthened. Finally, as with any bSSFP-based technique, off-resonance artifacts may be unacceptably severe in the presence of a ferromagnetic metallic implant (e.g., implantable cardioverter-defibrillator) and will be worse at high field (e.g., 3T). Finally, CT angiography might have provided a more accurate reference standard than gated CEMRA.

## 6. Conclusion

We found that using bT1RESS greatly prolongs the useful duration of blood pool contrast enhancement while improving angiographic image quality compared with standard CEMRA techniques. Although further study is needed, potential advantages for vascular imaging include eliminating the current requirement for first-pass imaging along with better reliability and accuracy for a wide range of cardiovascular applications.

## Funding

Research support: 10.13039/100000002NIH grants 1R01CA263091 and 1R21CA273280; Siemens Healthcare; Department of Radiology, NorthShore University HealthSystem.

## Author contributions

R.E.: participated in all aspects of the study and is the guarantor of study integrity. O.O.: assisted with data analysis and image interpretation. S.B.: assisted with image interpretation. A.P.: assisted with image interpretation. N.L.: assisted with subject scanning and data analysis. I.K.: assisted with pulse sequence implementation, statistical analysis, and manuscript review. All authors read and approved the manuscript.

## Ethics approval and consent

The study was approved by the hospital's institutional review board (IRB). Written informed consent was obtained for volunteers (n = 1). Waiver of consent was obtained for patients (n = 33) undergoing a clinically indicated cardiac or aortic MRI exam during which additional pulse sequences were obtained.

## Consent for publication

Consent for publication was provided under the approved IRB protocol. No protected health information for any subject is given in this manuscript.

## Declaration of competing interests

R.E.: research support and invention licensing agreement, Siemens Healthcare. Patent applications submitted. I.K.: patent applications submitted. There were no non-financial conflicts of interest for any of the authors.
